# Mapping Palliative Care Development in Asia Pacific: A Regional Milestone for Equity and System Reform

**DOI:** 10.1177/26892820251381245

**Published:** 2025-09-18

**Authors:** Laura Monzón Llamas, Vilma A. Tripodoro, Carlos Centeno

**Affiliations:** ^1^ ATLANTES Global Observatory of Palliative Care, Institute for Culture and Society, University of Navarra, Pamplona, Spain.; ^2^ IdiSNA, Institute for Health Research of Navarra, Pamplona, Spain.

**Keywords:** Asia Pacific, health equity, palliative care, service provision, WHO indicators

## Abstract

A new regional assessment of palliative care development in Asia Pacific provides a comprehensive overview of progress and gaps across 41 countries using standardized WHO indicators. Coordinated by the ATLANTES Global Observatory in partnership with the Asia Pacific Hospice Palliative Care Network, the initiative presents data on policy, education, service provision, access to medicines, and community engagement. Freely available online, the Atlas of Palliative Care in Asia Pacific supports equity-oriented system reform across diverse health contexts.

Despite increasing global recognition of palliative care (PC) as essential to universal health coverage (UHC), access remains uneven, especially in low- and middle-income countries. The Asia-Pacific region illustrates this paradox sharply. A recent regional assessment led by the ATLANTES Global Observatory of Palliative Care, in collaboration with the Asia Pacific Hospice Palliative Care Network (APHN), presents the first comprehensive mapping of PC development across the Asia Pacific region: The APHN Atlas of Palliative Care in Asia Pacific Regions 2025.^1^ Home to more than 4.3 billion people, the region has now been evaluated using standardized WHO indicators to produce a 390-page, evidence-based overview aligned with the WHO public health model.

The assessment drew on the contributions of collaborators across 41 countries. Although the data presented are still considered estimates, drawn from official documents and expert knowledge rather than exhaustive national registries, these experts were trained through an accredited program and followed a systematic methodology. Data were collected and validated across six key dimensions: policy, education, research, service integration, access to essential medicines, and community empowerment. This participatory approach ensured methodological rigor, regional ownership, and comparability between countries.

Key findings from the assessment paint a picture of stark imbalance. Most of the specialist PC services are concentrated in just six countries: Australia, Japan, South Korea, Malaysia, New Zealand, and Thailand. Only 24% of countries have integrated PC within health systems, leaving vast populations, particularly in rural or remote areas, underserved. One of the most critical gaps remains the limited availability of essential pain medications. Oral morphine, a cornerstone of effective pain management, is unavailable at the primary care level in 75% of territories. Only Australia, Japan, New Zealand, and Hong Kong SAR report consistent access to opioids in over 70% of rural settings. In many other countries, including the Philippines, Malaysia, Indonesia, and India, regulatory restrictions and insufficient training among health care providers continue to limit access.

Some countries offer compelling models of integration. Thailand, for instance, stands out for integrating PC into primary health services through regional coordination and national frameworks. In China, systemic investment has led to the establishment of more than 2280 specialist services, although access remains largely urban-centered. In Singapore, with PC education integrated into all undergraduate medical and nursing curricula, palliative medicine has been recognized as a subspecialty since 2007, and a dedicated Committee for the Integration of Palliative Care into Primary Care is actively working to expand the role of generalist PC.

Pediatric palliative care (PPC), while still largely hospital-based, is expanding in countries like Singapore, Hong Kong SAR, and Australia. As shown in Figure 1, specialized PPC services are limited or isolated in most countries and territories, with the majority at early stages of development and existing services predominantly concentrated in urban areas. Key challenges include the limited availability of trained PPC specialists, lack of integration into primary health care and community-based models, and restricted geographic coverage. Several countries also report the absence of formal training programs, inadequate policy frameworks, and weak referral mechanisms. Even in countries where PPC is more established, such as Australia and Japan, challenges with service integration and equitable distribution persist.

**FIG. 1.**
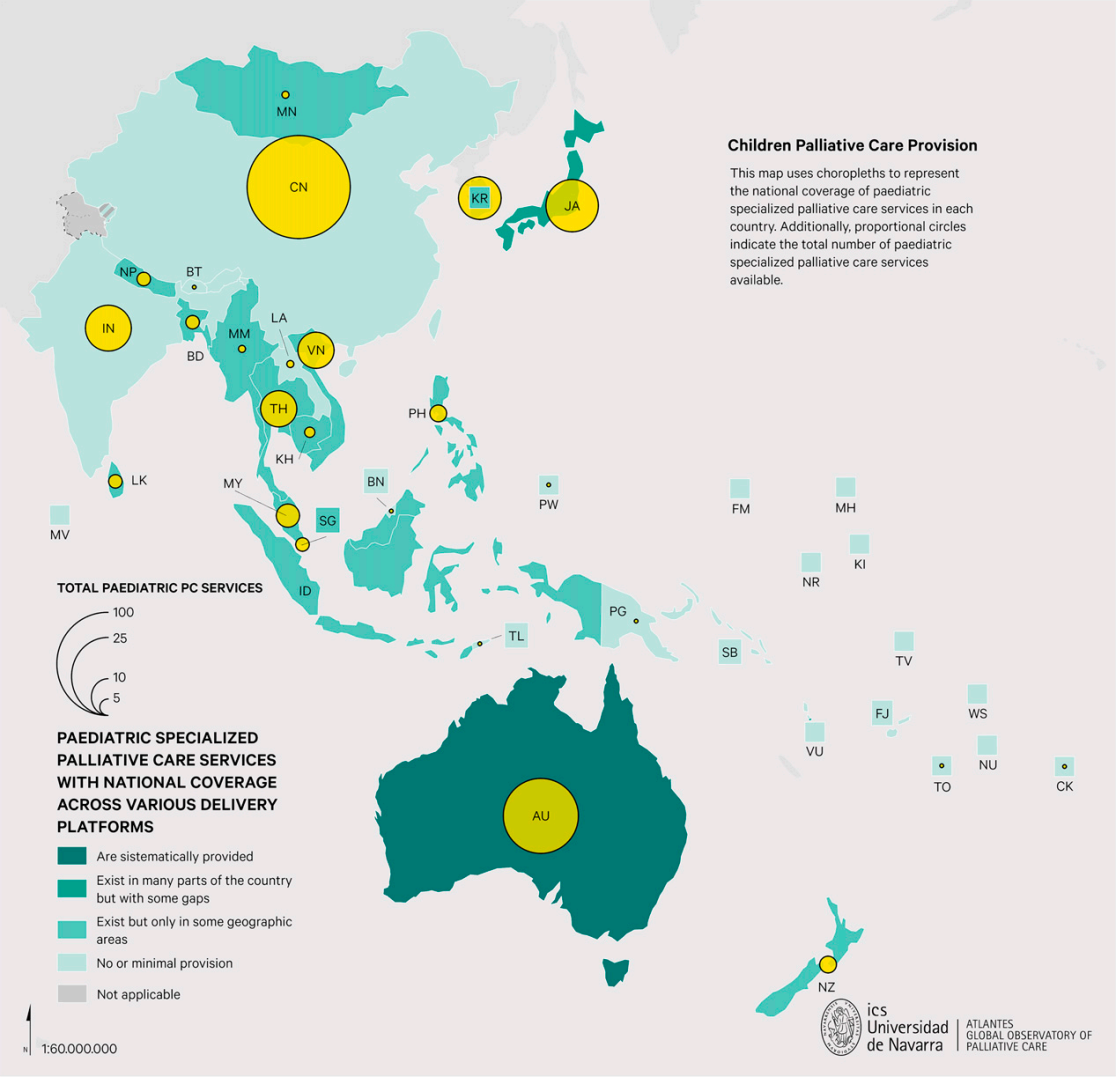
Pediatric palliative care provision by country. Adapted from the APHN Atlas of Palliative Care in the Asia Pacific Regions 2025.^1^ Pediatric Specialized Palliative Care Services with National Coverage Across Various Delivery Platforms. Reproduced with permission from the authors, ATLANTES Global Observatory of Palliative Care, University of Navarra.

Several key initiatives have contributed to advancing PC development in the Asia Pacific region. The 2015 Quality of Death Index by the Economist Intelligence Unit drew global attention to disparities in end-of-life care and helped initiate cross-country comparisons.^2^ Other efforts, including a multi-country survey from the APHN,^3^ along with bibliometric and indicator-based analyses of PC research and development,^4,5^ have deepened understanding of service availability, education, research, and policy contexts across the region. The APHN Atlas of Palliative Care in the Asia Pacific Regions 2025 builds on these foundations by offering the first comprehensive, region-wide assessment based on the WHO public health approach to PC development.

The open-access nature of the Atlas is central to its mission: the full text can be downloaded freely from the University of Navarra’s website, with an optional print edition available via Amazon. Additional materials such as a media kit and advocacy tools aim to support broader engagement by clinicians, policymakers, and health advocates across the region.

Beyond its function, the findings of the Atlas provide a foundation for strategic prioritization across key sectors. It is intended to serve Ministries of Health as a reliable source of evidence to inform the development or adjustment of national PC strategies, particularly in aligning services with UHC frameworks and health benefits packages. The identification of policy and service gaps highlights potential areas for targeted investment, particularly in capacity-building and system integration. The inclusion of PC in undergraduate medical, nursing, and allied health education emerges as a critical component for workforce preparedness. The document also underscores the importance of standardized measurement—not only for ongoing monitoring, resource planning, and evidence-informed advocacy, but also for contributing to more equitable and transparent PC systems across the Asia Pacific region.

In conclusion, this regional assessment establishes a vital new evidence base for advancing PC in Asia Pacific. It underscores critical disparities, showcases scalable models, and points toward practical opportunities for building more equitable, integrated systems of care. Equity—reflected in service access, resource distribution, and workforce capacity—emerges as a central theme of the Atlas, informing its structure, methodology, and policy recommendations.
